# Disruption of the c-Myc/miR-200b-3p/PRDX2 regulatory loop enhances tumor metastasis and chemotherapeutic resistance in colorectal cancer

**DOI:** 10.1186/s12967-017-1357-7

**Published:** 2017-12-19

**Authors:** Zhenbing Lv, Jinlai Wei, Wenxian You, Rong Wang, Jingkun Shang, Yongfu Xiong, Hua Yang, Xuanhua Yang, Zhongxue Fu

**Affiliations:** 1 Department of Gastrointestinal Surgery, The First Affiliated Hospital, Chongqing Medical University, Chongqing, 400016 China; 2 Department of Gastrointestinal Surgery, Nanchong Central Hospital, Nanchong, 637000 Sichuan China; 3 The Second Clinical School of North Sichuan Medical College, Nanchong, 637000 Sichuan China; 4 Department of Gastroenterology, The First Affiliated Hospital, Chongqing Medical University, Chongqing, 400016 China

**Keywords:** Colorectal cancer, c-Myc, miR-200b-3p, PRDX2, Chemotherapeutic resistance, Metastasis

## Abstract

**Background:**

Metastasis is a major threat to colorectal cancer (CRC) patients. We have reported that peroxiredoxin-2 (PRDX2) is associated with CRC invasion and metastasis. However, the mechanisms regulating PRDX2 expression remain unclear. We investigate whether microRNAs (miRNAs) regulate PRDX2 expression in CRC progression.

**Methods:**

Quantitative real-time polymerase chain reaction (qPCR) was used to measure microRNA-200b-3p (miR-200b-3p) expression. Immunohistochemistry (IHC) was performed to detect c-Myc and PRDX2 protein levels in CRC tissue samples (n = 97). Western blot was used to quantify PRDX2, c-Myc, AKT2/GSK3β pathway-associated proteins and epithelial-mesenchymal transition (EMT)-related proteins in CRC cells. Luciferase reporter assays were used to analyze the interaction between miR-200b-3p and 3′untranslated region (3′UTR) of PRDX2 mRNA and AKT2 mRNA as well as c-Myc and the miR-200b-3p promoter. Chromatin immunoprecipitation (ChIP) assay was used to evaluate binding of c-Myc to the miR-200b-3p promoter. Invasive assay and metastatic model were used to assess invasive and metastatic capacities of CRC cells in vitro and in vivo. Moreover, drug-induced apoptosis was measured by flow cytometry.

**Results:**

We found that miR-200b-3p was significantly downregulated, whereas c-Myc and PRDX2 were upregulated in metastatic CRC cells and CRC tissues compared to their counterparts. An inverse correlation existed between c-Myc and miR-200b-3p, and between miR-200b-3p and PRDX2. We also found that PRDX2 was a target of miR-200b-3p. Importantly, overexpression of nontargetable PRDX2 eliminated the suppressive effects of miR-200b-3p on proliferation, invasion, EMT, chemotherapeutic resistance and metastasis of CRC cells. Moreover, c-Myc bound to the promoter of miR-200b-3p and repressed its transcription. In turn, miR-200b-3p disrupted the stability of c-Myc protein by inducing c-Myc protein threonine 58 (T58) phosphorylation and serine 62 (S62) dephosphorylation via AKT2/GSK3β pathway.

**Conclusions:**

Our findings reveal that the c-Myc/miR-200b/PRDX2 loop regulates CRC progression and its disruption enhances tumor metastasis and chemotherapeutic resistance in CRC.

**Electronic supplementary material:**

The online version of this article (10.1186/s12967-017-1357-7) contains supplementary material, which is available to authorized users.

## Background

Colorectal cancer (CRC) is the second most common malignancy worldwide in females and the third in males, and one of the main causes of cancer death [1]. Metastasis is a major threat to patients’ survival and over a third of CRC patients will develop metastatic diseases [2]. However, little is known about the molecular mechanisms driving CRC metastasis. Uncovering metastasis-related molecules is important to develop new therapeutic strategies.

Peroxiredoxins (PRDXs) are a ubiquitous family of small thiol-dependent peroxidases that scavenge H_2_O_2_, alkyl hydroperoxides and peroxynitrite, and regulate various physiological and pathological processes, including carcinogenesis, tumor metastasis and development of drug resistance [3]. Peroxiredoxin-2 (PRDX2), an important member of peroxiredoxin family, has been reported to be aberrant expression in various cancers and exerts dual roles in cancer progression. On the one hand, a decreased expression of PRDX2 contributes to enhanced proliferation, motility and metastasis of melanoma cells [4]. Similarly, PRDX2 is epigenetically silenced and inhibits c-Myc-induced leukemogenesis in acute myeloid leukemia (AML) [5]. On the other hand, the PRDX2 has been found to be elevated in prostate, cervical, esophageal and vaginal cancers [6–9]. It has been reported that PRDX2 promotes proliferation of malignant B lymphocytes and therefore is regarded as a potential drug target to treat human Burkitt lymphoma [10]. PRDX2 also plays an essential role in maintaining stemness of hepatocellular carcinoma cells [11]. In addition, conflicting expression profiles and prognostic roles of PRDX2 are examined in CRCs [12–14]. We have reported that PRDX2 is upregulated in CRC tissues and correlates with CRC metastasis [14, 15]. Our most recent studies show that PRDX2 promotes vasculogenic mimicry formation by activating VEGFR2 [16] and contributes to maintain colorectal cancer stem cell-like properties [17], suggesting a possible implication of PRDX2 in regulating CRC invasion and metastasis. However, the molecular mechanisms have not yet been elucidated.

MicroRNAs (miRNAs) are small (19–24 nt), endogenous, and non-coding RNAs negatively regulating gene expression through binding to the 3′untranslated region (3′UTR) of their target mRNAs by complementary base pairing [18]. Growing studies show that miRNAs are involved in regulating cancer progression. MiR-539 inhibits prostate cancer proliferation, migration and invasion by targeting Sperm-associated antigen 5 [19]. MiR-200b enhances CRC cell proliferation [20], inhibits chemotherapy-induced EMT in tongue cancer cells [21], and blocks tumor metastasis through ZEB1-independent pathway in breast cancer [22]. However, there has been no published study to identify the potential miRNAs targeting PRDX2 in CRC progression.

In this study, we identified miR-200b-3p as a post-transcriptional regulator of PRDX2. We also found that c-Myc inhibited miR-200b-3p transcription and miR-200b-3p destabilized c-Myc. Moreover, we elucidated the contribution of the c-Myc/miR-200b/PRDX2 regulatory loop to the tumor EMT, metastasis, chemotherapeutic resistance and prognostic significance in CRC patients.

## Methods

### Cell lines and clinical samples

Human embryonal kidney 293T (293T) cells and CRC cell lines HCT116, Caco2, HT29, SW480, LoVo and SW620 were obtained from Shanghai Cell Bank of Type Culture Collection, China. All the cell lines were authenticated by short tandem repeat assay. The 293T cells were cultured in DMEM medium (Gibco, Gaithersburg, MD, USA) and the CRC cell lines in RPMI 1640 medium (Gibco), supplementing with 10% fetal bovine serum (FBS, HyClone, Logan, USA) at 37 °C in 5% CO_2_. Ninety-seven cases of CRC tissue samples and the paired normal colon mucosa (PNCM) tissues, including fresh tissue samples and paraffin-embedded tissue samples for each case, were obtained from patients who underwent CRC resection in Nanchong central hospital (Sichuan, China, Additional file 5: Table S1) between 2011 and 2012, with written informed consent. The fresh tissues were stored at – 80 °C until needed. All human experiments were approved by the ethics committee of the Nanchong central hospital.

### Transfection of CRC cells with lentiviral constructs

Lentiviral constructs expressing miR-200b-3p (Lenti-miR) or repressing miR-200b-3p (Lenti-Zip-miR) were purchased from Chongqing Maobai Technology Co., Ltd, China. Lentiviral constructs expressing the sequence coding (CDS) for protein of PRDX2 or c-Myc were purchased from Shanghai Genechem Co., Ltd., China.

5.0–7.0 × 10^4^ CRC cells were plated into 24-well plates (30–50% confluence) on the day before transfection. Lentiviral constructs were transfected into CRC cells at appropriate multiplicity of infection (MOI, 20 for SW480 cell and 30 for LoVo cell). Polybrene (Maobai, Chongqing, China) was used to enhance efficiency of transfection at the final concentration of 8 μL/mL. The infected cells were maintained with only DMEM medium for 12 h and then the medium was replaced with fresh DMEM medium supplementing with 10% FBS. The cells were harvested at 72 h after transfection for qPCR and western blotting validation.

### Quantitative real-time PCR (qPCR)

TRIzol method (Invitrogen, USA) was used to extract total RNA according to the manufacturer’s instructions. All-in-One miRNA qRT-PCR Detection Kit (GeneCopeia, USA) was used to detect miRNAs’ expression levels by the ABI 7500 System (Applied Biosystems, USA) according to the protocols. The expression levels of miRNAs were normalized to that of RNU6B by the 2-comparative Ct (2^−ΔΔCt^) method. The primers for miRNAs and RNU6B were purchased from GeneCopeia Company. The primers’ sequences cannot be disclosed because of a company owned patent.

### Cell proliferation assay

1 × 10^3^ cells were seeded into 96-well plates with three duplicate wells. The viable cells were detected by the Cell Counting Kit-8 (CCK-8, Dojindo,Kumamoto, Japan) method according to the manufacturer’s instructions using Thermo Scientific™ Varioskan™ LUX Multimode microplate reader (Thermo Scientific, USA) at 490 nm for 6 days.

### In vitro invasion assay

The invasive capacities of CRC cells were assessed using the established methods [23].

### Immunoblot (Western-blot) analysis

Total proteins of cell samples were extracted with lysis buffer and then quantified using the BCA method (KeyGen Biotech, Jiangsu, China). The lysate was diluted in SDS sample buffer (KeyGen Biotech) for SDS-polyacrylamide gelelectrophoresis (PAGE) and then transferred to polyvinylidene difluoride (PVDF) membranes (Roche Applied Sciences,USA). The membranes were immunoblotted at 4 °C overnight with anti-PRDX2 (Proteintech, USA), anti-c-Myc(Abcam, UK), anti-p-c-Myc(S62) (Abcam), anti-p-c-Myc(T58)(Abcam), anti-E-cadherin (Proteintech), anti-Vimentin (Proteintech), anti-N-cadherin (Proteintech), anti-GSK3β (Abcam), anti-p-GSK3β (Ser9) (Abcam), anti-AKT(1/2) (Abcam), anti-AKT1 (Abcam), anti-AKT2 (Abcam), anti-p-AKT2 (Ser474) (Abcam) and anti-GAPDH (Proteintech) antibodies at appropriate dilution concentration, followed by incubation using the appropriate second antibodies for 2 h. The bands were exposed using Pierce ECL Western Blotting Substrate (Thermo Scientific). Image J software was used to analyze the grey value of the interest protein.

### Immunohistochemistry (IHC)

Four-micrometer sections from CRC paraffin-embedded tissue samples were used to perform IHC staining by PV-9002 Detection Kits (ZSGB-Bio, Beijing, China) according to the manufacturer’s instructions. In brief, the sections were incubated in anti-PRDX2 antibody diluted to 1:500 or anti-c-Myc antibody 1:200 at 4 °C overnight. The results of IHC staining were evaluated by two experienced pathologist double-blindly, scoring 0–3 (Additional file 4: Figure S4a). The extent of IHC staining was presented by the average score.

### Drug treatment

For assessment of resistance of CRC cells to chemotherapy, CRC cells were treated with oxaliplatin (0, 0.625, 1.25, 2.5, 5, 10 and 20 μM) (Sigma-Aldrich, USA) for 72 h. The cell viabilities then were detected by CCK8 assay and the half-maximal inhibitory concentrations (IC50) were calculated using GraphPad Prism 5 software. For the drug-induced cytotoxicity analysis, CRC cells were treated with 5 μM oxaliplatin for 72 h. Subsequently, the cells were harvested and performed apoptosis detection by a FACS Aria cytometer (BD Bioscience) using the Annexin V-PE and 7-amino-actinomycin D (7-AAD) double-staining Apoptosis Detection kit (KeyGEN). For Tws119 (Selleck, USA) usage, CRC cells with 2 μM Tws119 treatment for 72 h were harvested for western blot analysis.

### Luciferase reporter assay

For the 3′UTR of PRDX2 and AKT2 mRNA luciferase reporter assays, the wild-type (wt) and mutant-type (mut) 3′UTR segments of PRDX2 or AKT2 containing putative miR-200b-3p binding site were synthesized and inserted into the pmirGLO vector (promaga, USA). The luciferase vectors and miR-200b-3p mimics (5′ uaauacugccugguaaugauga 3′, GenePharma,Shanghai, China) were cotransfected into 293T cells and LoVo cells using Lipofectamine 2000 (Invitrogen, USA). For the promoter of miR-200b-3p luciferase reporter assays, the CDS for protein of c-Myc were synthesized and inserted into the pcDNA3.1-EGFP vectors (Invitrogen), and the wt and mut promoters of miR-200b-3p were synthesized and inserted into the pGL3-promoter vectors (Promaga). The pGL3-promoter-miR-200b-3p vectors and pcDNA3. 1-c-Myc vectors were cotransfected with pRL-TK into 293T cells and SW480 cells using lipofectamine 2000. Luciferase activity was measured after 72 h by the Dual-Luciferase Reporter Assay Kit (Promega) according to the manufacturer’s instructions. The synthetic sequences were provided by Wuhan GeneCreate Biological Engineering Co., Ltd, China. All the recombinant vectors were validated by sequencing method (data not shown).

### Chromatin immunoprecipitation assay (ChIP)

The binding sites of c-Myc to the promoter of miR-200b-3p were detected using the chromatin immunoprecipitation assay kit (Millipore, Bedford, MA, USA) according to the manufacturer’s instructions. In brief, the protein-DNA complexes were precipitated in anti-c-Myc antibody diluted to 1:1000 at 4 °C overnight. Anti-IgG antibody was used as negative controls, while input as positive controls. The immunoprecipitated products were amplified by PCR using specific primers spanning the region of c-Myc putative binding sites. Primers are listed in Additional file 5: Table S2.

### Metastatic model establishment in nude mice

First, 1 × 10^6^ CRC cells with different treatments were subcutaneously injected into the female BALB/c nude mice (Beijing HFK Bioscience Co., LTD, Beijing China) aged 5 weeks for 4 weeks to generate subcutaneous tumors and then the mice were sacrificed. The schematic representation for positions of subcutaneous tumor after injection with CRC cells were shown in Additional file 1: Figure S1c. Second, the subcutaneous tumors were immediately isolated from the sacrificed mice and maintained in normal saline. Other cohort of female BALB/c nude mice (Beijing HFK Bioscience Co., LTD, Beijing China) aged 5 weeks were anesthetized and their cecum were exteriorized by laparotomy. The subcutaneous tumors were cut into the same size (about 30 mm^3^) to be embedded into the mesentery at the distal end of cecum, and then the cecum was restored prior to surgical sutures. Six weeks later, the mice were sacrificed and their intestines, livers and lungs were removed for biopsy. LEICA DM600 upright microscope (Germany) was used to observe metastatic lesions after HE staining. The nude mice were maintained under specific pathogen-free (SPF) conditions in the experimental animal center of Chongqing medical university. All animal experiments were performed according to protocols approved by the institutional animal care and use committee at the Chongqing medical university.

### Statistical analysis

All statistical analyses were performed using SPSS 13.0 software. Quantitative results are presented as the mean ± SD. Paired student t test was used to analyze statistical significance of gene or protein expression between CRC and PNCM tissues. One-way ANOVA or the independent samples t test was used to analyze statistical significance of ones among or between groups. Pearson’s correlation analysis was used to evaluate the correlation. Kaplan–Meier method was used to analyze statistical significance of overall survival and plot survival curves. A *p* value < 0.05 was considered to be statistically significant.

## Results

### MiR-200b-3p is a post-transcriptional regulator of PRDX2

**Fig. 1:**
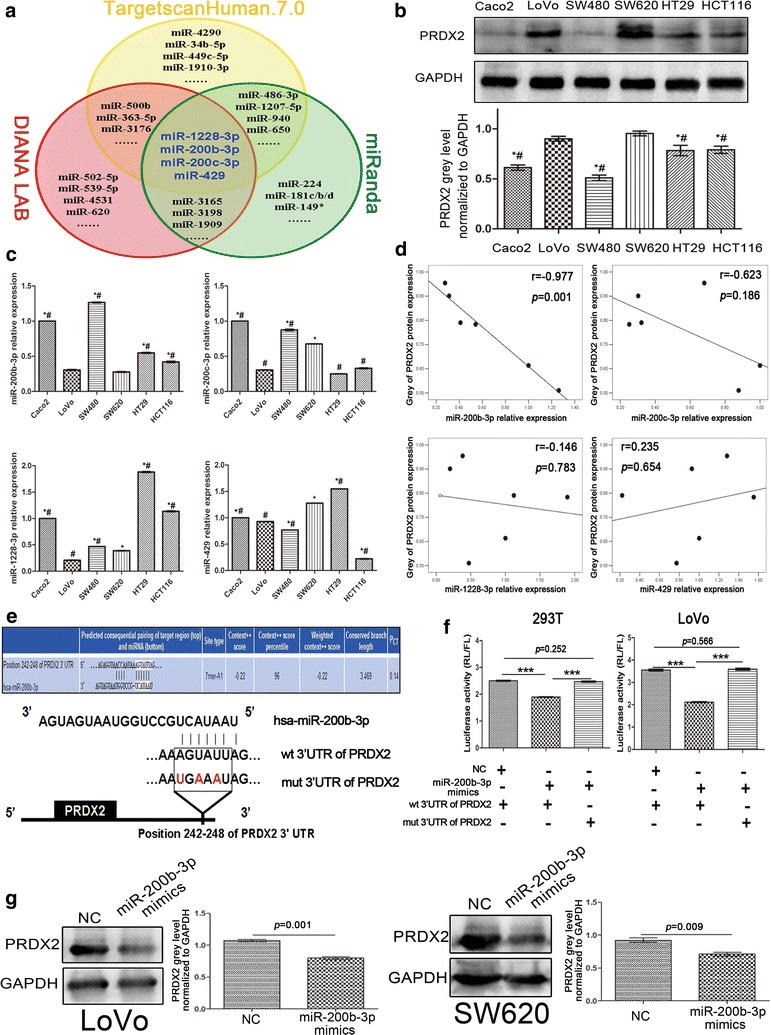
PRDX2 is a target of miR-200b-3p. **a** Wenn diagram of prediction for possible miRNAs targeting PRDX2 by three bioinformatic databases. **b** PRDX2 protein levels in six CRC cell lines were detected by western blot and quantified by Image J software. The grey value of PRDX2 was normalized to that of the corresponding GAPDH. **c** Expression levels of miR-200b-3p, miR-200c-3p, miR-1228-3p and miR-429 were detected in six CRC cell lines by qPCR. The relative expression levels were normalized to Caco2 cell (*p < 0.05 compared to LoVo cell,^#^ *p* < 0.05 compared to SW620 cell). **d** Pearson correlation analysis of PRDX2 protein levels with miR-200b-3p, miR-200c-3p, miR-1228-3p and miR-429 expression in six CRC cell lines. **e** Predictive binding sites and mutant sites of miR-200b-3p to 3′UTR of PRDX2 mRNA. **f** The luciferase activities of wild-type and mutant-type pmirGLO-3′UTRs of PRDX2 in 293T and LoVo cells after transfection of miR-200b-3p mimics (***p < 0.001, * RL* Ranilla luciferase,* FL* Firefly luciferase). **g** PRDX2 protein levels were detected by western blot in SW620 and LoVo cells after transfection of miR-200b-3p mimics. The grey value of PRDX2 was normalized to that of the corresponding GAPDH

To identify miRNAs targeting PRDX2, bioinformatic algorithms, including TargetscanHuman.7.0, DIANA LAB and miRanda, were used to screen for miRNA candidates. As shown in Fig. 1a, miR-1228-3p, miR-200b-3p, miR-200c-3p and miR-429 were agreed by all three algorithms. To further narrow down miRNA candidates, we measured each miRNA expression level and PRDX2 protein level in six CRC cell lines with different metastatic potentials. Western-blot analysis showed that PRDX2 protein level was higher in the metastatic cell lines (SW620 and LoVo) than in the pre-invasive cell lines (SW480, Caco2, HT29 and HCT116) (Fig. 1b). Conversely, qPCR showed that miR-1228-3p and miR-200b-3p had lower expression levels in the metastatic cell lines than in the pre-invasive cell lines (Fig. 1c). Pearson correlation analysis showed that only miR-200b-3p expression level was significantly inverse correlation with PRDX2 protein level(*r* = − 0.977, *p* = 0.001, Fig. 1d), suggesting that miR-200b-3p might be a negative regulator of PRDX2.

To confirm whether miR-200b-3p regulates PRDX2 negatively, we constructed pmirGLO-3′UTRs of PRDX2 luciferase vectors (Fig. 1e). Reporter assays showed that ectopic miR-200b-3p expression dramatically suppressed the luciferase activity of wild-type (wt) PRDX2 3′UTR in 293T cells and LoVo cells, while it did not suppress the luciferase activity of mutant-type (mut) PRDX2 3′UTR (Fig. 1f). Consistent with results of reporter assays, we found ectopic miR-200b-3p reduced PRDX2 protein level (Fig. 1g). These results showed that miR-200b-3p targeted PRDX2 3′UTR and disrupted its protein expression.

### MiR-200b-3p represses oncogenic properties of CRC cells by targeting PRDX2 in vitro

**Fig. 2:**
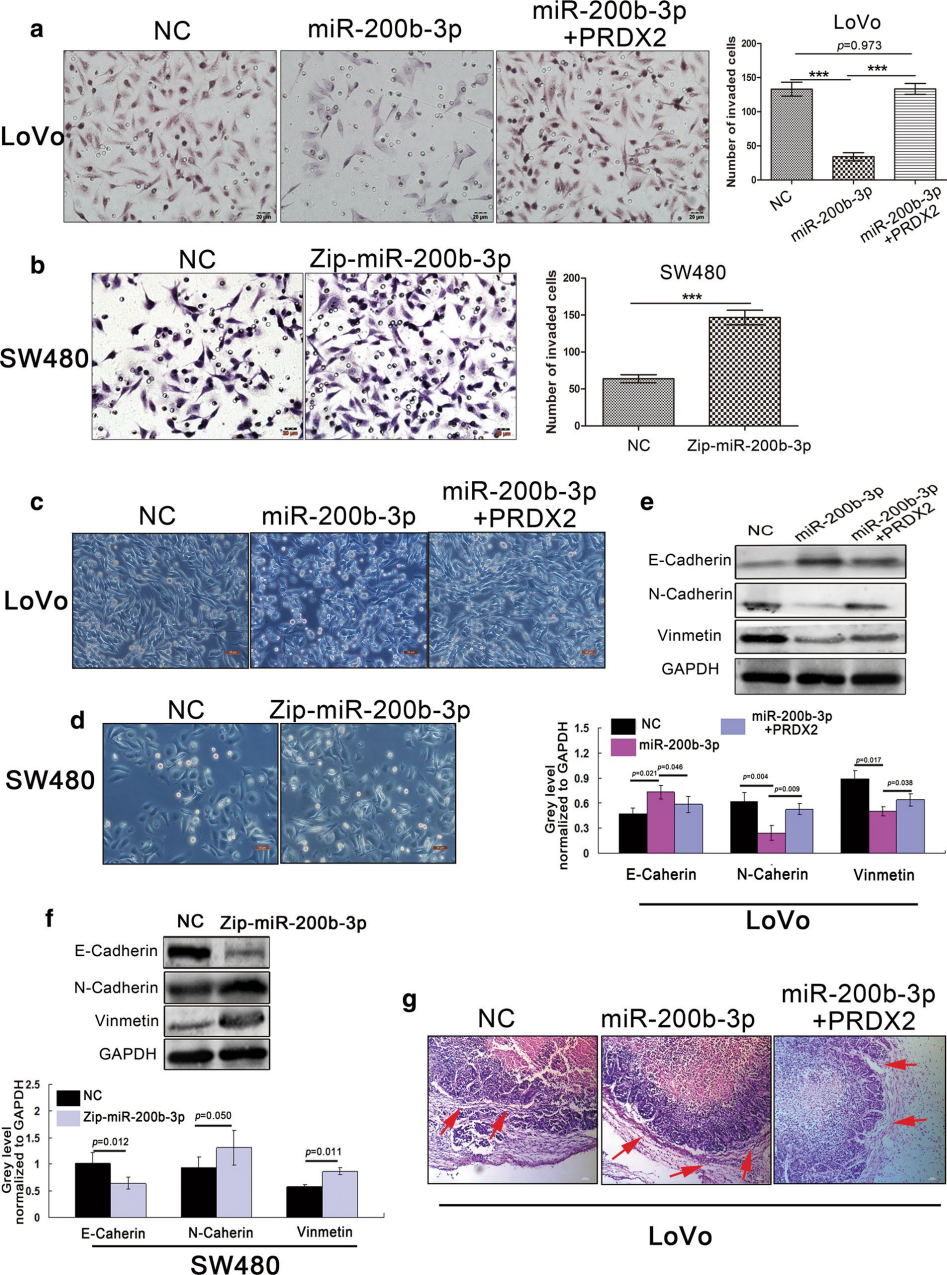
MiR-200b-3p inhibits CRC invasion and EMT by targeting PRDX2 in vitro and in vivo. **a** The effect of overexpression of miR-200b-3p and co-expression of miR-200b-3p and nontargetable PRDX2 on the invasion of LoVo cells by Boyden chamber. Scale bars represent 20 μm (***p < 0.001). **b** The effect of downregulation of miR-200b-3p on the invasion of SW480 cells by Boyden chamber. Scale bars represent 20 μm (***p < 0.001). **c** Morphological changes of LoVo cells were observed under the microscope after overexpression of miR-200b-3p and co-expression of miR-200b-3p and nontargetable PRDX2. Scale bars represent 50 μm. **d** Morphological changes of SW480 cells were observed under the microscope after downregulation of miR-200b-3p. Scale bars represent 50 μm. **e** Key EMT-related markers were detected by western blot in LoVo cells after overexpression of miR-200b-3p and co-expression of miR-200b-3p and nontargetable PRDX2. The grey value of the protein was normalized to that of the corresponding GAPDH. **f** Key EMT-related markers were by Western blot in SW480 cells after downregulation of miR-200b-3p. The grey value of the protein was normalized to that of the corresponding GAPDH. **g** HE staining for local invasion of subcutaneous tumors derived from LoVo/NC, LoVo/miR and LoVo/miR + PRDX2 cells. Red arrows point at false fibrous membrane. Scale bars represent 50 μm

To investigate the effects of miR-200b-3p, we established LoVo/miR cells stably expressing miR-200b-3p, LoVo/miR + PRDX2 cells stably co-expressing miR-200b-3p and nontargetable PRDX2 and SW480/Zip-miR cells stably silencing miR-200b-3p (Additional file 1: Figure S1a). CCK8 proliferation assays showed that miR-200b-3p overexpression inhibited CRC cell proliferation, whereas miR-200b-3p silencing promoted CRC cell proliferation (Additional file 1: Figure S1b). Similarly, transwell invasive assays showed that miR-200b-3p overexpression dramatically inhibited invasive behavior of LoVo cells (Fig. 2a), while miR-200b-3p silencing showed the opposite effect in SW480 cells (Fig. 2b). Noticeably, miR-200b-3p overexpression reduced frequencies of cells with fibroblastic or spindle-like morphology and concomitantly increased frequencies of cobblestone-like cells (Fig. 2c). In contrast, miR-200b-3p silencing showed the opposite effect (Fig. 2d). This suggested that miR-200b-3p might inhibit CRC cell EMT. Further supporting this notion, miR-200b-3p overexpression increased the expression of the epithelial marker E-cadherin and decreased the expression of the mesenchymal markers N-cadherin and vimentin, and vice versa (Fig. 2e, f). Importantly, these suppressive effects of miR-200b-3p on malignant behaviors of LoVo cells were substantially weakened by the nontargetable PRDX2 (Fig. 2a, c, e), suggesting that PRDX2 is a functional target of miR-200b-3p in regulating biological behaviors of CRC cells in vitro.

### MiR-200b-3p suppresses growth, invasion and metastasis of CRC cells by targeting PRDX2 in vivo

**Fig. 3:**
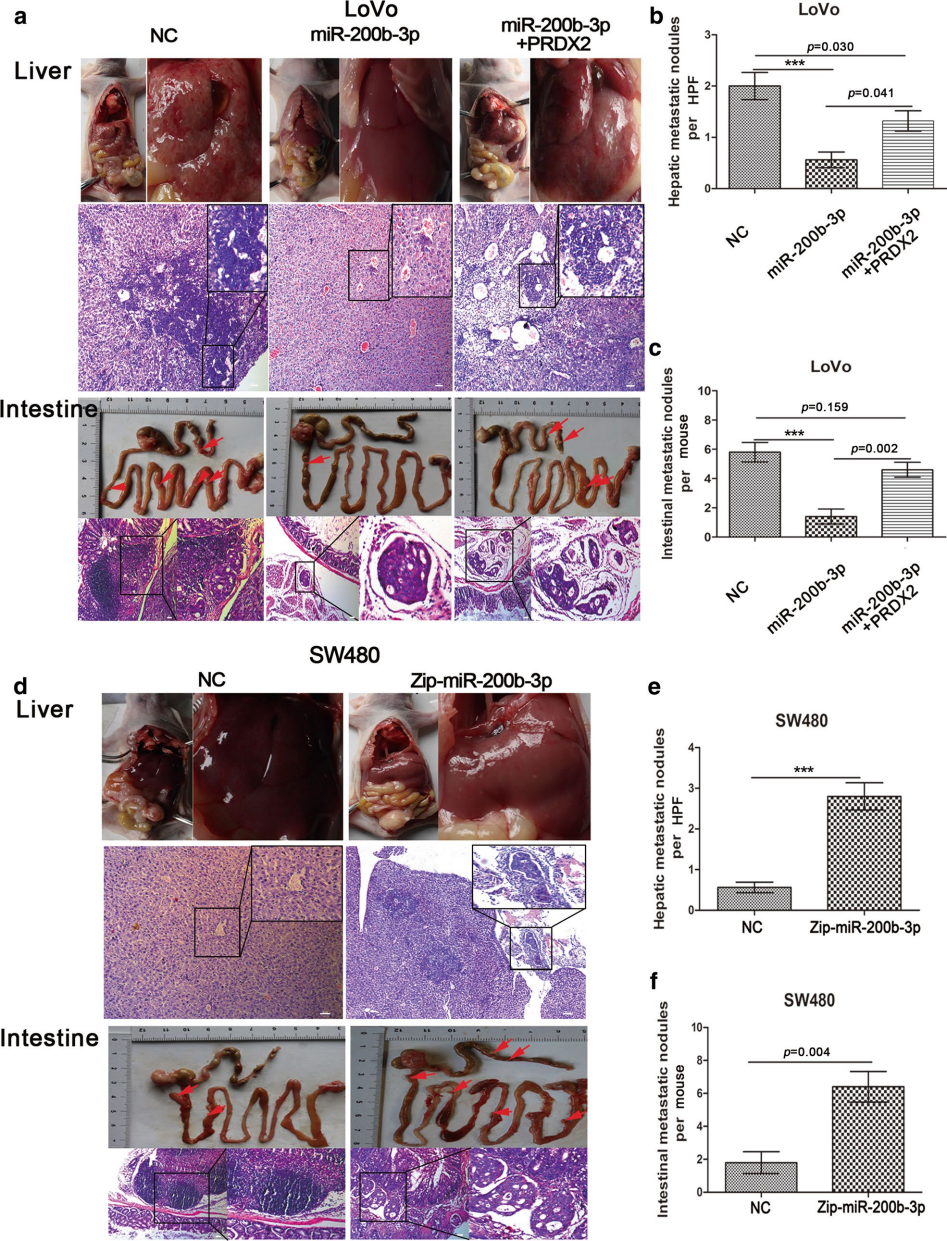
MiR-200b-3p inhibits CRC cells’ metastatic capacity by targeting PRDX2 in vivo. **a** Intestinal and hepatic metastatic nodules after subcutaneous tumors derived from LoVo/NC, LoVo/miR and LoVo/miR + PRDX2 cells were transplanted in the mesentery at the distal end of cecum in mice (n = 5) for 6 weeks. Red arrows point at potential metastatic nodules in intestines. Scale bars represent 50 μm. **b**, **c** The number of hepatic metastatic nodules (**b**) or intestinal metastatic nodules (**c**) of mice with tumors derived from LoVo/NC, LoVo/miR and LoVo/miR + PRDX2 cells. The number of hepatic metastatic nodules per mouse was counted under the microscope, with five high power fields (HPF) observation (***p < 0.001). **d** Intestinal and hepatic metastatic nodules after subcutaneous tumors derived from SW480/NC and SW480/Zip-miR cells were transplanted in the mesentery at the distal end of cecum in mice (n = 5) for 6 weeks. Red arrows point at potential metastatic nodules in intestines. Scale bars represent 50 μm. **e**, **f** The number of hepatic metastatic nodules (**e**) or intestinal metastatic nodules (**f**) of mice with tumors derived from SW480/NC and SW480/Zip-miR cells. The number of hepatic metastatic nodules per mouse was counted under the microscope, with five HPF observation (***p < 0.001)

Based on our in vitro results, we speculated that miR-200b-3p might be involved in regulating biological behaviors of CRC cells in vivo. To test it, we injected LoVo/miR, LoVo/miR + PRDX2 and SW480/Zip-miR cells subcutaneously in mice. We found that mice with LoVo/miR cells injection displayed inhibited tumor growth (Additional file 1: Figure S1c, d), while mice with SW480/Zip-miR cells injection showed the opposite effect (Additional file 1: Figure S1e). Intriguingly, the subcutaneous tumors derived from LoVo/miR cells were well encapsulated in false fibrous membrane (Fig. 2g), suggesting that miR-200b-3p could inhibit invasive behavior of CRC cell in vivo. To model metastatic process of CRC, we transplanted the subcutaneous tumor in the mesentery at the distal end of caecum of nude mice. We found that mice with miR-200b-3p overexpressing tumors developed fewer metastatic nodules in intestine and liver than control mice with tumors of similar size (Fig. 3a–c). In contrast, mice with miR-200b-3p silencing tumors showed the more metastatic nodules than control mice (Fig. 3d–f). Unexpectedly, a lung metastasis was observed under microscope in a mouse with miR-200b-3p silencing tumor (Additional file 1: Figure S1f). Importantly, the nontargetable PRDX2 rescued the miR-200b-3p-induced repression of CRC cell growth, invasion and metastasis in vivo (Figs. 2g, 3a–c and Additional file 1: Figure S1c, d), suggesting that PRDX2 is sufficient to mediate the effects of miR-200b-3p on regulating biological behaviors of CRC cells in vivo.

### MiR-200b-3p is transcriptionally regulated by c-Myc

**Fig. 4:**
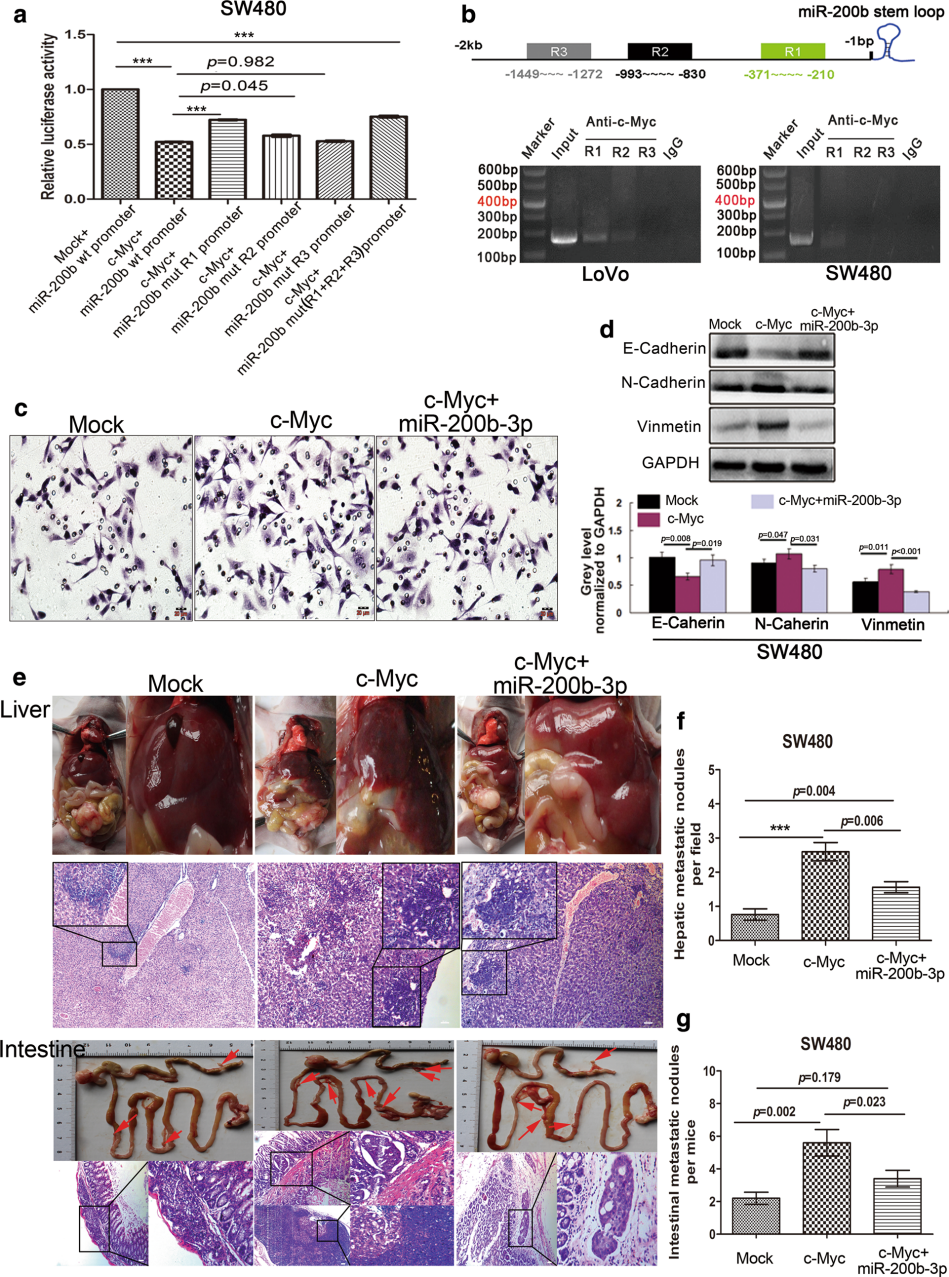
c-Myc promotes invasion, EMT and metastasis of CRC cell by repressing miR-200b-3p. **a** The luciferase activity of PGL3-promoter-miR-200b-3p constructs of wild type and mutant types after transfection of pCDA3.1-c-Myc constructs in SW480 cells. **b** ChIP assay in LoVo and SW480 cells. Three regions containing putative c-Myc binding sites in the R1, R2 and R3 of miR-200b-3p promoter were amplified by PCR with specific primers. IgG was used to be negative controls, while input positive controls. **c** The effect of overexpression of c-Myc and co-expression of c-Myc and miR-200b-3p on the invasion of SW480 cells by Boyden chamber. Scale bars represent 20 μm. **d** Key EMT-related markers were detected by western blot in SW480 cells after overexpression of c-Myc and co-expression of c-Myc and miR-200b-3p. The grey value of the protein was normalized to that of the corresponding GAPDH. **e** Intestinal and hepatic metastatic nodules after subcutaneous tumors derived from SW480/Mock, SW480/c-Myc and sw480/c-Myc+miR cells were transplanted in the mesentery at the distal end of cecum in mice (n = 5) for 6 weeks. Red arrows point at potential metastatic nodules in intestines. Scale bars represent 50 μm. **f**, **g** The number of hepatic metastatic nodules (**f**) or intestinal metastatic nodules (**g**) of mice with tumors derived from SW480/Mock, SW480/c-Myc and sw480/c-Myc + miR cells. The number of hepatic metastatic nodules per mouse were counted under the microscope, with five HPF observation (***p < 0.001)

Growing studies have shown the regulation of miRNAs’ expression by transcription factors [23, 24]. As described in the previous study [24], we chose 2-kb region directly upstream of miR-200b-3p stem loop as the putative promoter. Possible binding motifs of c-Myc were found in the putative promoter of miR-200b-3p using Consite (http://consite.genereg.net/) on line database. An inverse correlation between c-Myc protein and miR-200b-3p was observed in six CRC cells (r = − 0.908, *p* = 0.012, Fig. 1c and Additional file 2: Figure S2a). Luciferase report assay showed that c-Myc strongly repressed the activity of the miR-200b-3p promoter in 293T and SW480 cells (Fig. 4a and Additional file 2: Figure S2b). ChIP assay showed that c-Myc bound to the region 1 (R1, − 371 to − 210 bp) and region 2 (R2, − 993 to − 830 bp) of the miR-200b-3p promoter (Fig. 4b). Further supporting the role of c-Myc in regulating miR-200b-3p expression, we found that overexpression of c-Myc inhibited miR-200b-3p expression level (Additional file 2: Figure S2c) in CRC cells. These results indicated that c-Myc bound to the promoter of miR-200b-3p and suppressed its expression.

### c-Myc strengthens malignant capacities of CRC cells by repressing miR-200b-3p in vitro and in vivo

To determine whether miR-200b-3p was responsible for the effects of c-Myc on CRC progression, we established SW480/c-Myc cells stably expressing c-Myc and SW480/c-Myc + miR cells stably co-expressing c-Myc and miR-200b-3p (Additional file 2: Figure S2d). We found that overexpression of c-Myc in SW480 cells enhanced cell invasiveness and EMT in vitro, which was dampened by re-expression of miR-200b-3p (Fig. 4c, d and Additional file 3: Figure S3a, b). In accord with in vitro results, in vivo metastatic assays demonstrated that overexpression of c-Myc strongly promoted proliferation and metastasis of SW480 cells, while the promoting effects could be rescued by re-expression of miR-200b-3p, at least partly (Fig. 4e–g and Additional file 3: Figure S3c, d). These results showed c-Myc/miR-200b-3p signal pathway was involved in regulating aggressive behaviors of CRC cells in vitro and in vivo.

### MiR-200b-3p counteracts c-Myc and disrupts its protein stability by inhibiting AKT2/GSK3β pathway

**Fig. 5:**
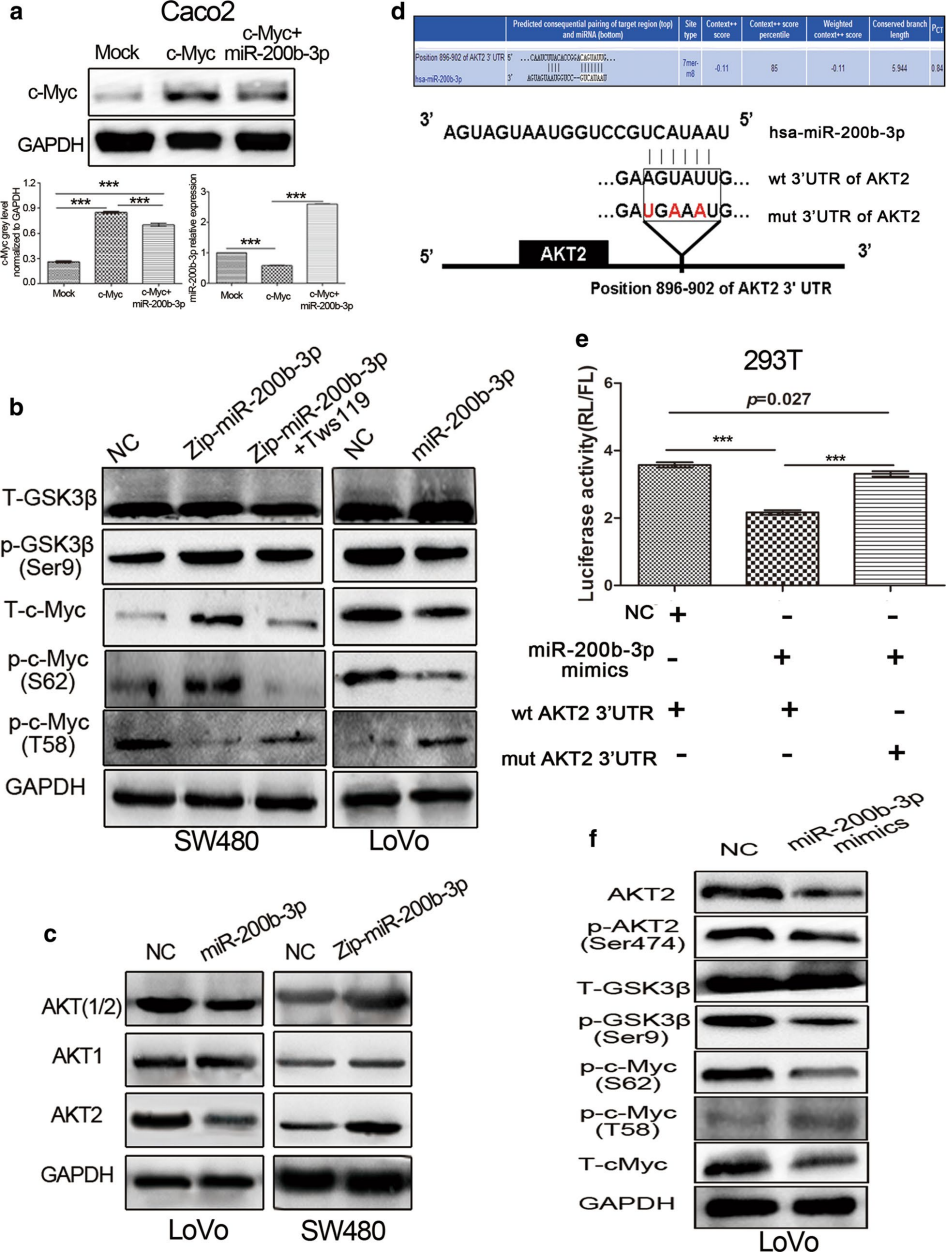
MiR-200b-3p disrupts stability of c-Myc protein by inhibiting AKT2/GSK3β pathway. **a** Detection of c-Myc protein by western blot and miR-200b-3p by qPCR after overexpression of c-Myc and co-expression of c-Myc and miR-200b-3p in Caco2 cells. The grey value of c-Myc was normalized to that of the corresponding GAPDH (***p < 0.001). **b** Total GSK3β, p-GSK3β (Ser9), total c-Myc, p-c-Myc (S62) and p-c-Myc (T58) protein levels were detected by western blot in LoVo/miR, SW480/Zip-miR and SW480/Zip-miR cells with Tws119 treatment for 72 h. **c** AKT (1/2), AKT1 and AKT2 were detected by western blot in LoVo/miR and SW480/Zip-miR cells. **d** Predictive binding sites and mutant sites of miR-200b-3p to 3′UTR of AKT2 mRNA. **e** The luciferase activities of wild-type and mutant-type pmirGLO-3′UTRs of AKT2 mRNA in 293T cells after transfection of miR-200b-3p mimics (***p < 0.001). **f** AKT2, p-AKT2 (Ser474), total GSK3β, p-GSK3β (Ser9), total c-Myc, p-c-Myc (S62) and p-c-Myc (T58) protein were detected by western blot after transfection of miR-200b-3p mimics

Our findings reveal that the c-Myc/miR-200b-3p/PRDX2 regulatory axis plays an important role in regulating CRC progression. Interestingly, we noticed that c-Myc protein level in SW480/c-Myc cells was decreased after re-expression of miR-200b-3p (Additional file 2: Figure S2d). The same phenomenon was also observed in Caco2 cells (Fig. 5a), suggesting that miR-200b-3p was involved in regulation of c-Myc protein expression. Based on bioinformatic analysis, the possibility that direct effect of miR-200b-3p on c-Myc by binding to its 3′UTR was ruled out (data not shown). Recent studies show that altered phosphorylation status of c-Myc protein at S62 and T58 residues affects the protein stability [25, 26]. The phosphorylation at T58 residue initiates c-Myc ubiquitylation, which contributes to the protein degradation. GSK3β has been reported to play an important role in c-Myc protein phosphorylation modification [27]. Therefore, we hypothesized GSK3β could mediate the effect of miR-200b-3p on regulation of c-Myc protein stability. As expected, our results demonstrated that miR-200b-3p silencing increased Ser9 phosphorylation of GSK3β without altering total GSK3β protein level (Fig. 5b). MiR-200b-3p silencing also decreased T58 phosphorylation but increased S62 phosphorylation of c-Myc protein (Fig. 5b). Conversely, miR-200b-3p overexpression showed the opposite changes of phosphorylation in GSK-3β and c-Myc proteins (Fig. 5b). Noticeably, following treating for 72 h with Tws119, a selective GSK-3β inhibitor, we found that the increased Ser9 phosphorylation of GSK3β and S62 phosphorylation of c-Myc, and the decreased T58 phosphorylation of c-Myc induced by miR-200b-3p silencing were well abolished (Fig. 5b).

Unexpectedly, miR-200b-3p overexpression decreased total Protein Kinase B 1/2 (AKT1/2) protein levels, particularly total AKT2 protein level, and vice versa (Fig. 5c). AKT2 represses GSK3β activity by increasing the phosphorylation level of GSK3β protein at Ser9 residues [28], and has been reported to be upregulated in CRC tissues compared to normal colon mucosa and promote tumor progression [29, 30]. The opposite effects between AKT2 and miR-200b-3p on CRC progression made us investigate the potential interaction between them. Indeed, bioinformatic analysis showed a conserved complementary sequence of miR-200b-3p present in AKT2 mRNA 3′UTR, suggesting AKT2 was a potential target of miR-200b-3p (Fig. 5d). Luciferase reporter assay showed ectopic miR-200b-3p expression inhibited the activity of wt 3′UTR of AKT2, but failed to inhibit the activity of mut 3′UTR of AKT2 in 293T cells (Fig. 5e). Moreover, we found that miR-200b-3p overexpression decreased total AKT2 protein and AKT2 Ser474 phosphorylation levels, which led to deceased Ser9 phosphorylation of GSK3β, increased T58 phosphorylation and deceased S62 phosphorylation of c-Myc, and a decrease in c-Myc protein level (Fig. 5f). Our results demonstrated that miR-200b-3p disrupted c-Myc protein stability through inhibiting AKT2/GSK3β pathway, suggesting that a regulatory loop consisting of the c-Myc/miR-200b-3p/PRDX2 axis and AKT2/GSK3β pathway was involved in regulating CRC progression.

### The expression of c-Myc, miR-200b-3p and PRDX2 is disrupted in human CRC tissues and their expression levels are associated with clinicopathological features and survival of CRC patients

**Fig. 6:**
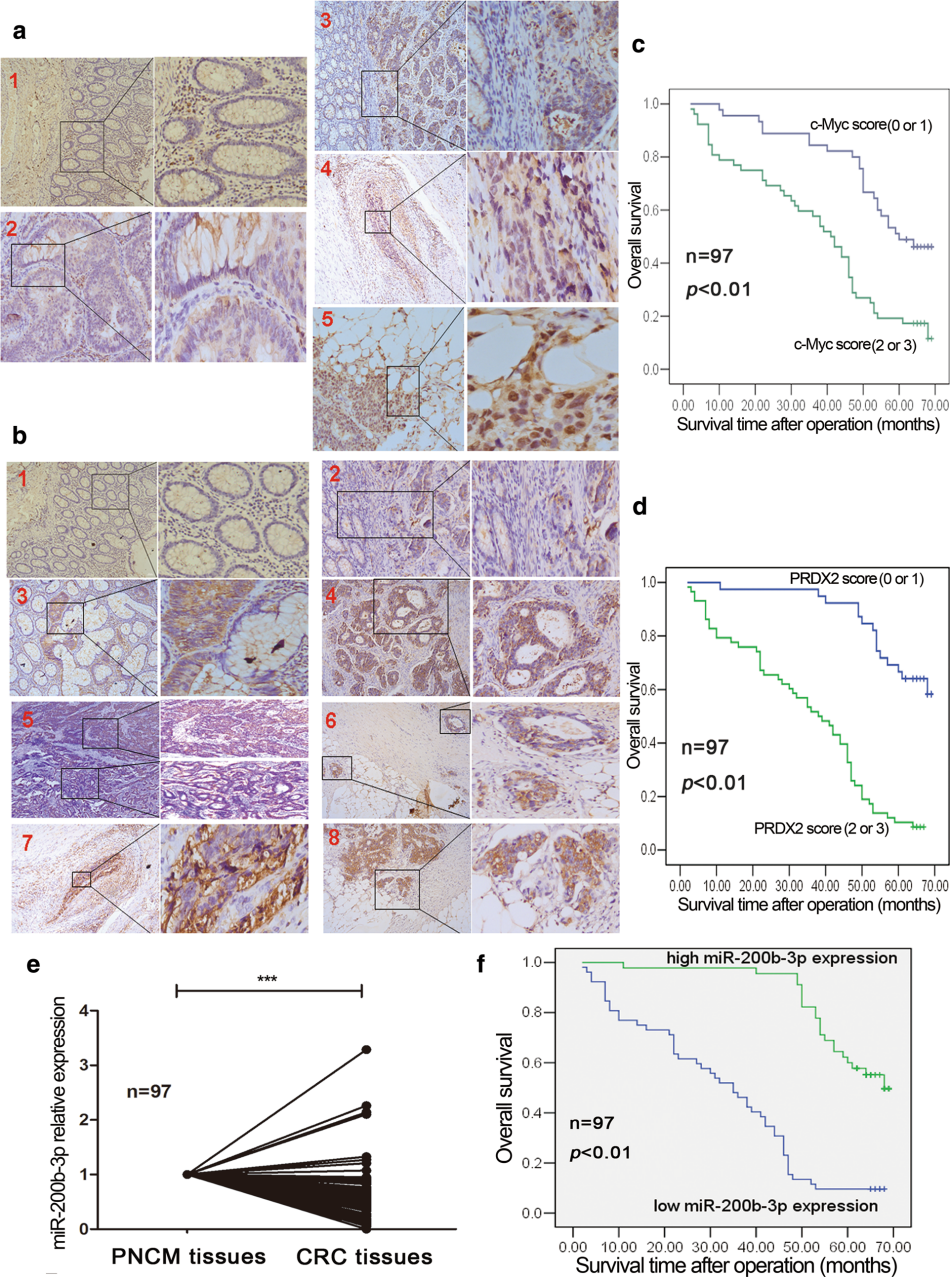
Expression profiles of c-Myc, miR-200b-3p and PRDX2 in CRC tissue samples and their clinical significances. **a** c-Myc protein expression profile was detected by IHC in paraffin-embedded CRC tissue samples (n = 97). **a**1 The expression levels of c-Myc in adjacent normal colon mucosa (×100, ×200). **a**2 The expression levels of c-Myc in intramucosal carcinoma and normal colon mucosa (×100, ×400). **a**3 The expression levels of c-Myc in submucosa carcinoma and normal colon mucosa (×100, ×200). **a**4 The expression levels of c-Myc in carcinoma infiltrated vessel of muscular layer (×100, ×400). **a**5 The expression levels of c-Myc in carcinoma infiltrated serosa layer (×100, ×400). **b** PRDX2 protein expression profile was detected by IHC in paraffin-embedded CRC tissue samples (n = 97). **b**1 The expression levels of PRDX2 in adjacent normal colon mucosa (×100, ×200). **b**2 The expression levels of PRDX2 in submucosa carcinoma and normal colon mucosa (×100, ×200). **b**3 The expression levels of PRDX2 in intramucosal carcinoma and normal colon mucosa (×100, ×200). **b**4 The expression levels of PRDX2 in carcinoma infiltrated muscular layer (×100, ×200). **b**5 The expression levels of PRDX2 in carcinoma with moderate-differentiation (lower) and poor-differentiation (upper) (×50, ×100). **b**6 The expression levels of PRDX2 in carcinoma infiltrated muscular layer (upper) and subserosa layer (lower) (×100, ×200). **b**7 The expression levels of PRDX2 in carcinoma infiltrated vessel of muscular layer (×100, ×400). **b**8 The expression levels of PRDX2 in carcinoma infiltrated serosa layer (×100, ×200). **c**, **d** Kaplan-Meier survival analysis for CRC patients with c-Myc score (0, 1) and (2, 3) (**c**) or PRDX2 score (0, 1) and (2, 3) (**d**). **e** Relative miR-200b-3p expression profile was detected by qPCR in paired fresh CRC tissue samples (n = 97). The miR-200b-3p expression of each CRC tissue was normalized to that of the PNCM tissue (***p < 0.001). **f** Kaplan–Meier survival analysis for CRC patients with high and low miR-200b-3p expression

Having uncovered the important involvement of the c-Myc/miR-200b-3p/PRDX2 regulatory loop in CRC progression, we speculated that their expression profiles might be perturbed in human CRC tissues. To test it, we assessed c-Myc, miR-200b-3p and PRDX2 expression profiles in 97 cases of paired CRC samples. IHC results showed c-Myc and PRDX2 protein levels were frequently higher in CRC tissues than in adjacent normal mucosa tissues (Fig. 6a, b and Additional file 4: Figure S4b). These samples were then scored based on c-Myc and PRDX2 staining extent (Additional file 4: Figure S4a) for survival analysis. As shown in Fig. 6c, d, the overall survival of CRC patients with low c-Myc or PRDX2 score (0 or 1) was significantly longer than that of CRC patients with high c-Myc or PRDX2 score (2 or 3). qPCR analysis showed that miR-200b-3p expression was downregulated in most CRC tissues compared to in PNCM tissues (Fig. 6e) and its expression level was negatively correlated with c-Myc and PRDX2 protein levels (Additional file 4: Figure S4c, d). To evaluate the effect of miR-200b-3p on overall survival of CRC patients, we stratified all cases into two groups (low miR-200b-3p expression: less than 60% expression of the PNCM tissue, high miR-200b-3p expression: more than and equal to 60% expression of the PNCM tissue) according to their miR-200b-3p level (Additional file 4: Figure S4e). As shown in Fig. 6f, CRC patients with high miR-200b-3p expression had a longer overall survival than CRC patients with low miR-200b-3p expression. Moreover, we found that c-Myc, miR-200b-3p and PRDX2 expression levels were well associated with tumor differentiation, size and pathological Tumor-Node-Metastasis (pTNM) stage (Additional file 5: Table S1).

### Disruption of the c-Myc/miR-200b-3p/PRDX2 regulatory loop enhances chemotherapeutic resistance of CRC cells

**Fig. 7:**
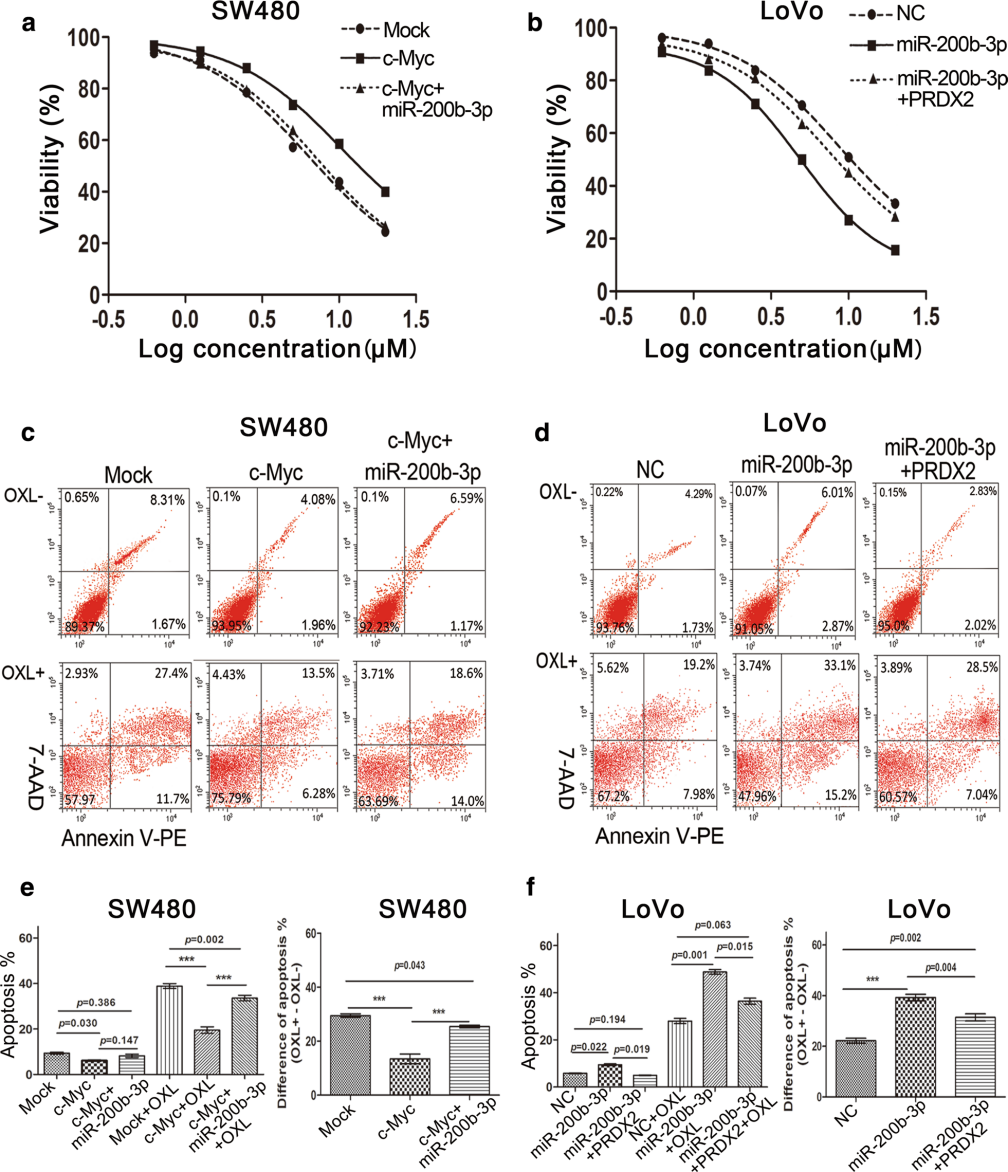
Disruption of the c-Myc/miR-200b-3p/PRDX2 regulatory loop enhances chemotherapeutic resistance of CRC cells. **a** c-Myc overexpression enhanced the resistance of SW480 cells to oxaliplatin, while the enhanced effect was dampened by miR-200b-3p partly. **b** miR-200b-3p overexpression reduced the resistance of LoVo cells to oxaliplatin, while the reduced effect was reversed by nontargetable PRDX2 partly. **c** c-Myc overexpression repressed oxaliplatin-induced apoptosis in SW480 cells, while the repressive effect was reversed by miR-200b-3p partly. **d** miR-200b-3p overexpression enhanced oxaliplatin-induced apoptosis in LoVo cells, while the enhanced effect was abolished by nontargetale PRDX2 partly. **e**, **f** The oxaliplatin-induced apoptosis was statistically analyzed in LoVo cells (**e**) or in SW480 cells (**f**) among different treatment groups

Chemotherapeutic resistance is associated with metastasis [21]. We proposed that disruption of the c-Myc/miR-200b-3p/PRDX2 loop might enhance resistance of CRC cells to chemotherapy. As expected, We found that the half inhibitory concentration (IC50) of SW480/c-Myc cells to oxaliplatin (OXL), the most commonly used drug in CRC chemotherapy, was dramatically higher than that of the control SW480 cells (10.39 μM vs. 6.365 μM), while re-expression of miR-200b-3p partly abolished the increased resistance to oxaliplatin (7.054 μM vs. 10.39 μM), as shown in Fig. 7a. We also found that LoVo/miR cells had decreased resistance to oxaliplatin compared to the control LoVo cells (4.859 μM vs. 8.521 μM), while the decreased resistance was restored by nontargetable PRDX2 (4.859 μM vs. 7.325 μM, Fig. 7b). Moreover, we investigated the effect of the loop on drug-induced cytotoxicity. After CRC cells were treated with 5 μM of oxaliplatin for 72 h, we found that overexpression of c-Myc reduced apoptosis ratios compared to the control, while re-expression of miR-200b-3p abrogated the reduced effects (Fig. 7c, e). We also found that miR-200b-3p overexpression increased apoptosis ratios, while the increased effect was rescued by nontargetable PRDX2 (Fig. 7d, f). These findings indicated that perturbation of the c-Myc/miR-200b/PRDX2 regulatory loop increased chemotherapeutic resistance.

## Discussion

In this study, we identify miR-200b-3p as a direct posttranscriptional regulator of PRDX2 and clarify that miR-200b-3p inhibits aggressive behaviors of CRC cells by inhibiting PRDX2. Noticeably, we demonstrate that downregulation of miR-200b-3p in CRC is partly due to transcriptional regulation by c-Myc, and provide the first evidence that miR-200b-3p counteracts c-Myc. Additionally, we show that c-Myc, miR-200b-3p and PRDX2 expression profiles are disrupted in CRC tissues and their expression levels are correlated with disease phenotypes and overall survival of CRC patients.

Previous studies have shown that PRDX2 is upregulated in CRC tissues and correlates with CRC metastasis, and overexpression of PRDX2 promotes CRC cell proliferation and invasion [12, 14–17]. However, the mechanisms responsible for upregulation of PRDX2 in CRC are unclear. Several studies have reported that PRDX2 is silenced by the promoter hyper-methylation in melanoma [4] and miR-200c in lung cancer [31], although these findings are not sufficient to interpret the mechanisms underlying the upregulation of PRDX2 in CRC. In this study, we used several miRNA target prediction databases to analyze potential miRNAs targeting PRDX2. MiR-200b-3p was identified by all databases. We further found that PRDX2 protein level was inversely correlated with miR-200b-3p expression level in CRC cell lines. Ectopic miR-200b-3p expression suppressed PRDX2 protein level. These results were verified by luciferase report assay. These observations demonstrate that miR-200b-3p is a post-transcriptional regulator of PRDX2.

MiR-200b-3p, also known as miR-200b, is an important member of miR-200 family, a tumor-suppressive miRNA family [32, 33]. MiR-200b-3p has been reported to inhibit proliferation, EMT, invasion and metastasis in cancers [34–39]. Consist with these studies, we found that miR-200b-3p repressed malignant phenotypes in vitro and metastasis in vivo of CRC cells. Interestingly, two studies in CRC reported that miR-200b-3p stimulates tumor growth by targeting CDKN1B and negatively regulating p27/kip1 in TGFBR2-null CRC [40], and miR-200b-3p overexpression promotes CRC cell proliferation by targeting reversion-inducing cysteine-rich protein with Kazal motifs (RECK) [20]. These findings seem to be in contradiction with suppressive effects of miR-200b-3p on CRC progression in our studies. Also, the conflicting effects of miR-200b-3p have been observed in cervical cancer [39, 41]. It well known that miRNA exerts its roles though directly inhibiting its targets, which may be implicated in cancer progression, some as tumor-promoting genes and others as tumor-suppressive genes. The functional discrepancy of miR-200b-3p among studies may thus result from different functional targets that play predominant roles under specific circumstances or in a given cancer, suggesting that miR-200b-3p is a context-dependent effecter [42]. Similarly, we found that miR-200b-3p was dramatically lower in CRC tissues than in PNCM tissues, while previous report shows that miR-200b-3p level is significantly elevated in CRC patients’ plasma, especially in stage III–IV patients’ plasma compared to in controls [43]. The opposite expression profiles in CRC may be attributed to tissue-specific expression pattern of miR-200b-3p, which just supports that miR-200b-3p is a context-dependent molecular.

**Fig. 8:**
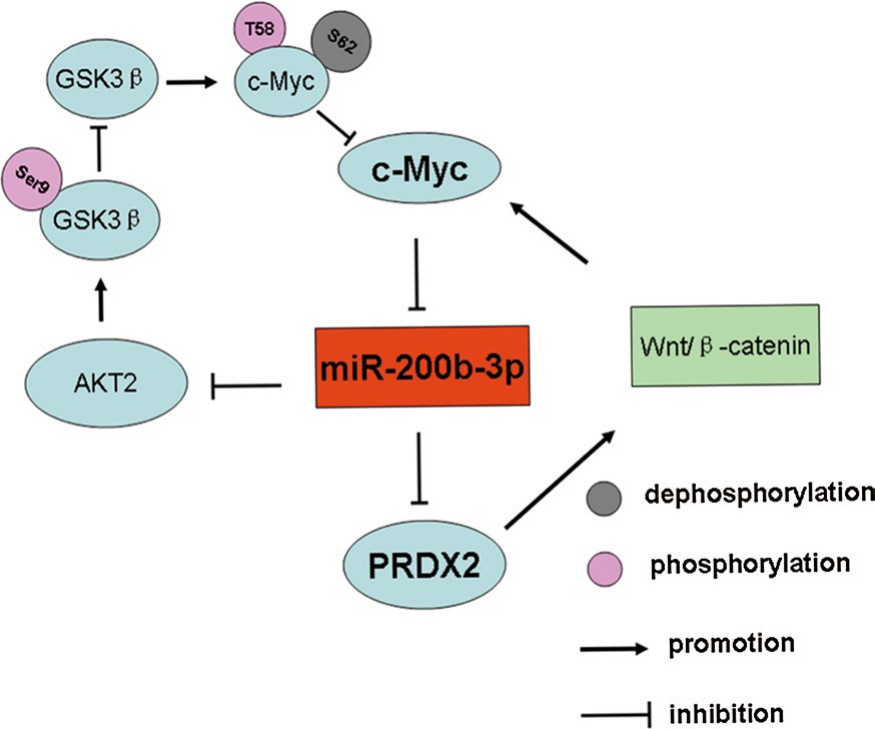
Schematic representation of the c-Myc/miR-200b-3p/PRDX2 regulatory loop. c-Myc inhibits miR-200b-3p expression, and miR-200b-3p suppresses PRDX2 protein level. In turn, on the one hand, miR-200b-3p reduces c-Myc protein level by disrupting T58 phosphorylation and S62 phosphorylation status of c-Myc protein via suppressing AKT2/GSK3β pathway. On the other hand, miR-200b-3p inhibits c-Myc expression by repressing the activity of wnt/β-cateinin pathway though directly suppressing PRDX2

Despite the extensive efforts to understand the contribution of miR-200b-3p to cancer behaviors, the mediators responsible for miR-200b-3p downregulation in CRC remain unidentified. In our study, ChIP and luciferase report assay showed that the transcription factor c-Myc bound to the promoter of miR-200b-3p and suppressed its expression. This is consistent with a previous study in endometrial carcinoma cell lines [44]. In addition, we found that miR-200b-3p reversed the promoting effects of c-Myc on CRC cell aggressive behaviors, indicating that miR-200b-3p is a functional mediator of c-Myc in CRC. Interestingly, we have reported in previous study that PRDX2 silencing contributes to decreased c-Myc expression by downregulating Wnt/β-catenin pathway [15], suggesting miR-200b-3p could potentially counteract c-Myc by regulating PRDX2. Noticeably, our current study showed that miR-200b-3p reduced c-Myc protein expression at the post-translational level by increasing T58 phosphorylation level and deceasing S62 phosphorylation level via inhibiting the activity of AKT2/GSK3β pathway, which provides new evidence supporting that miR-200b-3p counteracts c-Myc. To sum up, c-Myc inhibits miR-200b-3p expression, followed by an increase in PRDX2 protein. In turn, decreased miR-200b-3p expression increases the activity of AKT2/GSK3β pathway, which contributes to stabilize c-Myc protein. Further, increased PRDX2 protein elevated c-Myc protein level by upregulating Wnt/β-catenin pathway. Therefore, we identified a novel self-reinforcing regulatory feedback loop in CRC mainly consisting of c-Myc, miR-200b-3p and PRDX2, as shown in Fig. 8. Moreover, we found the loop was disrupted in human CRC tissue samples, forcefully supporting that the self-reinforcing regulatory feedback loop is involved in CRC progression. However, further studies are required to clarify that miR-200b-3p exerts its roles by AKT2/GSK3β/c-Myc pathway in CRC progression, making us more fully understand the self-reinforcing regulatory loop.

Since tumor chemotherapeutic resistance is associated with metastasis [21], we assessed contribution of the c-Myc/miR-200b-3p/PRDX2 regulatory loop to chemotherapeutic resistance of CRC cells. We found that c-Myc inhibited, and miR-200b-3p induced, apoptosis of CRC cells. These results may provide a mechanism by which we understand the effects of c-Myc and miR-200b-3p on growth of CRC cells, although it is not sufficient to explain differences of apoptosis ratio between CRC cells with and without oxaliplatin treatment (Mock group, 29.50 ± 1.14% vs. c-Myc group, 13.45 ± 3.06%, *p* < 0.001. NC group, 22.19 ± 1.67% vs. miR-200b-3p group, 39.26 ± 2.27%, *p* < 0.001). At least a reasonable explanation is that c-Myc reduced, and miR-200b-3p enhanced oxaliplatin-induced cytotoxicity. As expected, the suppressive effect of c-Myc on oxaliplatin-induced cytotoxicity was abolished by re-expression of miR-200b-3p, which was reversed by nontargetable PRDX2. These results suggest that disruption of the c-Myc/miR-200b-3p/PRDX2 regulatory loop contributes to enhanced chemotherapeutic resistance of CRC.

## Conclusions

In summary, we identify a novel regulatory loop consisting of c-Myc, miR-200b-3p, AKT2/GSK3β pathway, PRDX2 and Wnt/β-catenin pathway in CRC progression. The present study reveals that disruption of the c-Myc/miR-200b-3p/PRDX2 regulatory loop enhances tumor metastasis and chemotherapeutic resistance of CRC cells, and contributes to unfavorable outcome of CRC patients. Understanding the molecular mechanisms of the c-Myc/miR-200b-3p/PRDX2 regulatory loop should help identify targets to impede CRC progression and metastasis.

## Additional files

**Additional file 1: Figure S1.** MiR-200b-3p inhibits CRC growth, invasion and metastasis in vitro and in vivo. (a) The transfection efficiency of lentiviral vectors expressing miR-200b-3p and co-expressing miR-200b-3p and nontargetable PRDX2 in LoVo cells or silencing miR-200b-3p in SW480cells were measured by western blot and qPCR. The grey value of PRDX2 was normalized to that of the corresponding GAPDH. (*** *p* < 0.001). (b) The effect of miR-200b-3p on the proliferation in LoVo and SW480 cells by CCK8 assay in vitro. (c) The schematic representation for positions of subcutaneous tumor formation after injection with LoVo/miR and LoVo/miR+PRDX2 cells in the nude mice. (d, e) LoVo/NC, LoVo/miR and LoVo/miR+PRDX2 cells (1 × 10^6^) (d) or SW480/NC and SW480/Zip-miR (1 × 10^6^) (e) were subcutaneously injected into the nude mice (n = 5) for four weeks and the isolated subcutaneous tumors were observed with naked eyes. (f) Lung metastasis was observed under the microscope. Black arrows point at metastatic lesion in lung. Scale bars represent 50 μm.

**Additional file 2: Figure S2.** c-Myc represses transcriptionally miR-200b-3p expression. (a) Protein expression levels of c-Myc in six CRC cell lines were detected by western blot and quantified by Image J software. The grey value of c-Myc was normalized to that of the corresponding GAPDH. Pearson correlation analysis for miR-200b-3p and c-Myc protein levels in six CRC cell lines. (b) The luciferase activity of PGL3-promoter-miR-200b-3p constructs of wild type and mutant types after transfection of pCDA3.1-c-Myc constructs in 293T cells (*** *p* < 0.001). (c) miR-200b-3p was detected by qPCR in SW480 and Caco2 cells after transfection of pCDA3.1-c-Myc constructs. The c-Myc protein was measured to assess the transfection efficiency of pCDA3.1-c-Myc constructs by western blot (*** *p* < 0.001). (d) The transfection efficiency of lentiviral vectors expressing c-Myc and co-expressing c-Myc and miR-200b-3p were measured by western blot and qPCR in SW480 cells. The grey value of c-Myc was normalized to that of the corresponding GAPDH. (*** *p* < 0.001).

**Additional file 3: Figure S3.** c-Myc represses growth, invasion and EMT of CRC cell by regulating miR-200b-3p. (a) Morphological changes of SW480 cells were observed under the microscope after overexpression of c-Myc and co-expression of c-Myc and miR-200b-3p. Scale bars represent 50 μm. (b) The number of invaded cells was counted under the microscope, with five HPF observation (*** p < 0.001). (c) SW480/Mock, SW480/c-Myc and sw480/c-Myc+miR cells (1 × 10^6^) were subcutaneously injected into the nude mice (n = 5) for four weeks and the isolated subcutaneous tumors was observed with naked eyes. (d) HE staining for local invasion of subcutaneous tumors derived from SW480/Mock, SW480/c-Myc and SW480/c-Myc+miR cells. Red arrows point at false fibrous membrane. Scale bars represent 50 μm.

**Additional file 4: Figure S4.** MiR-200b-3p is inversely correlated with c-Myc and PRDX2. (a) IHC staining for c-Myc and PRDX2 protein in CRC tissue samples. The c-Myc protein is mainly expressed in cell nucleus, whereas PRDX2 in cytoplasm. The scores (0, 1, 2 and 3) of the c-Myc and PRDX2 are based on their staining extents. (b) c-Myc and PRDX2 protein expression levels were frequently upregulated in CRC tissues compared to in PNCM tissues. (c, d) Inverse correlation of miR-200b-3p expression with c-Myc protein level (c), and with PRDX2 protein level (d) in CRC tissues. (e) The percentage of low and high miR-200b-3p expression in 97 cases of CRC tissues was presented in the pie chart.

**Additional file 5: Table S1.** The expression levels of c-Myc, PRDX2 and miR-200b-3p correlate with disease phenotypes of CRC patients. **Table S2.** Primer sequences for PCR amplification.

## Abbreviations

CRC: colorectal cancer
PRDX2: peroxiredoxin-2
miRNA: microRNA
miR-200b-3p: microRNA-200b-3p
qPCR: quantitative real-time polymerase chain reaction
IHC: immunohistochemistry
AKT: protein kinase B
EMT: epithelial-mesenchymal transition
3′UTR: 3′untranslated region
ChIP: chromatin immunoprecipitation
RL: Ranilla luciferase
FL: Firefly luciferase
T58: threonine 58
S62: serine 62
Wt: wild-type
Mut: mutant-type
PNCM: paired normal colon mucosa
CCK8: cell counting kit-8
AML: acute myeloid leukemia
TNM: tumor-node-metastasis
OXL: oxaliplatin
