# Supplementary figures and images for: Disruption of the c-Myc/miR-200b-3p/PRDX2 regulatory loop enhances tumor metastasis and chemotherapeutic resistance in colorectal cancer
Source: J Transl Med. 2017 Dec 19;15:257. doi: 10.1186/s12967-017-1357-7 (PMC5735915; doi:10.1186/s12967-017-1357-7)

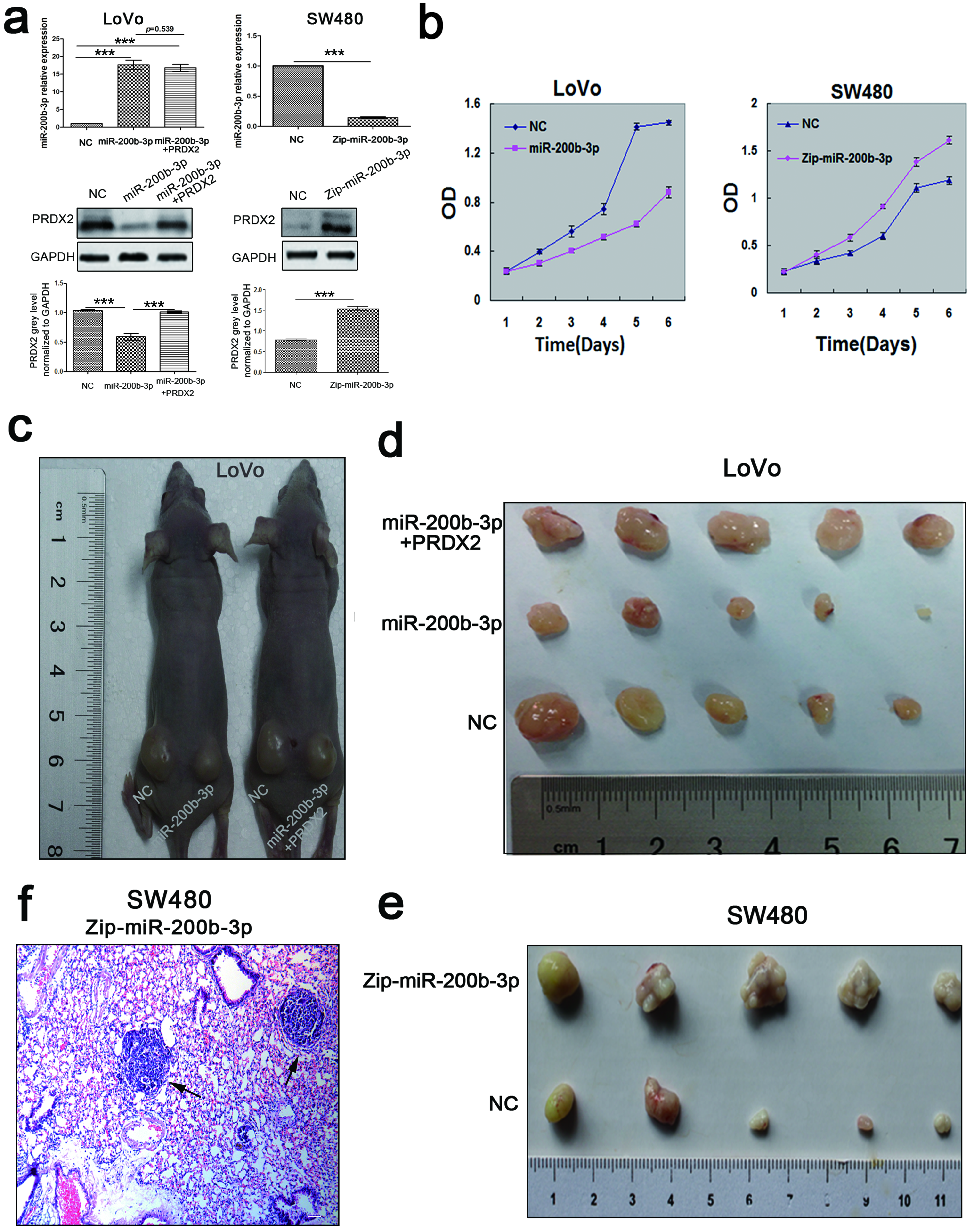

Supplement: Supplementary file 1 — Additional file 1: Figure S1. MiR-200b-3p inhibits CRC growth, invasion and metastasis in vitro and in vivo. (a) The transfection efficiency of lentiviral vectors expressing miR-200b-3p and co-expressing miR-200b-3p and nontargetable PRDX2 in LoVo cells or silencing miR-200b-3p in SW480cells were measured by western blot and qPCR. The grey value of PRDX2 was normalized to that of the corresponding GAPDH. (*** p < 0.001). (b) The effect of miR-200b-3p on the proliferation in LoVo and SW480 cells by CCK8 assay in vitro. (c) The schematic representation for positions of subcutaneous tumor formation after injection with LoVo/miR and LoVo/miR+PRDX2 cells in the nude mice. (d, e) LoVo/NC, LoVo/miR and LoVo/miR+PRDX2 cells (1 × 10^6^) (d) or SW480/NC and SW480/Zip-miR (1 × 10^6^) (e) were subcutaneously injected into the nude mice (n = 5) for four weeks and the isolated subcutaneous tumors were observed with naked eyes. (f) Lung metastasis was observed under the microscope. Black arrows point at metastatic lesion in lung. Scale bars represent 50 μm. [file 12967_2017_1357_MOESM1_ESM.tif]

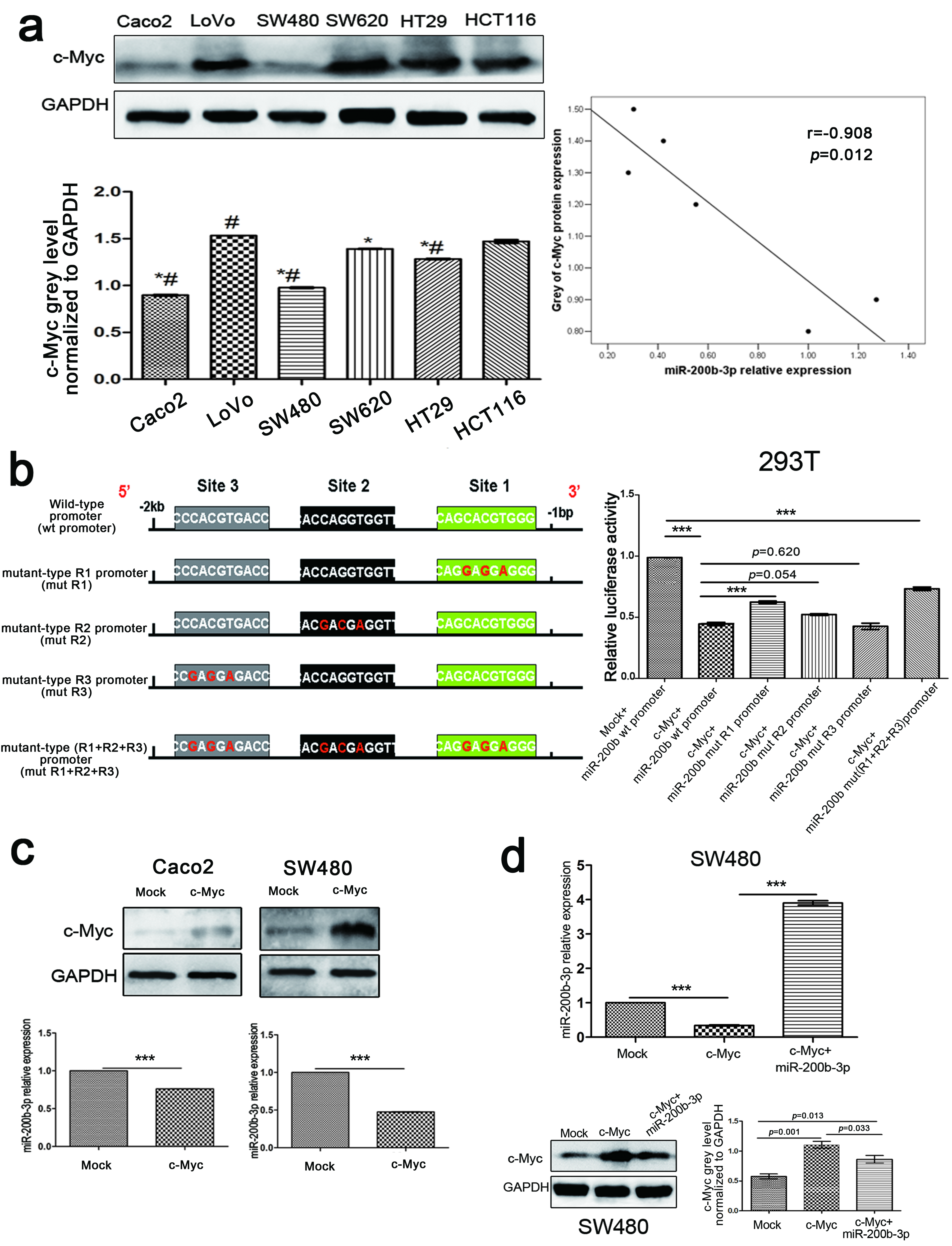

Supplement: Supplementary file 2 — Additional file 2: Figure S2. c-Myc represses transcriptionally miR-200b-3p expression. (a) Protein expression levels of c-Myc in six CRC cell lines were detected by western blot and quantified by Image J software. The grey value of c-Myc was normalized to that of the corresponding GAPDH. Pearson correlation analysis for miR-200b-3p and c-Myc protein levels in six CRC cell lines. (b) The luciferase activity of PGL3-promoter-miR-200b-3p constructs of wild type and mutant types after transfection of pCDA3.1-c-Myc constructs in 293T cells (*** p < 0.001). (c) miR-200b-3p was detected by qPCR in SW480 and Caco2 cells after transfection of pCDA3.1-c-Myc constructs. The c-Myc protein was measured to assess the transfection efficiency of pCDA3.1-c-Myc constructs by western blot (*** p < 0.001). (d) The transfection efficiency of lentiviral vectors expressing c-Myc and co-expressing c-Myc and miR-200b-3p were measured by western blot and qPCR in SW480 cells. The grey value of c-Myc was normalized to that of the corresponding GAPDH. (*** p < 0.001). [file 12967_2017_1357_MOESM2_ESM.tif]

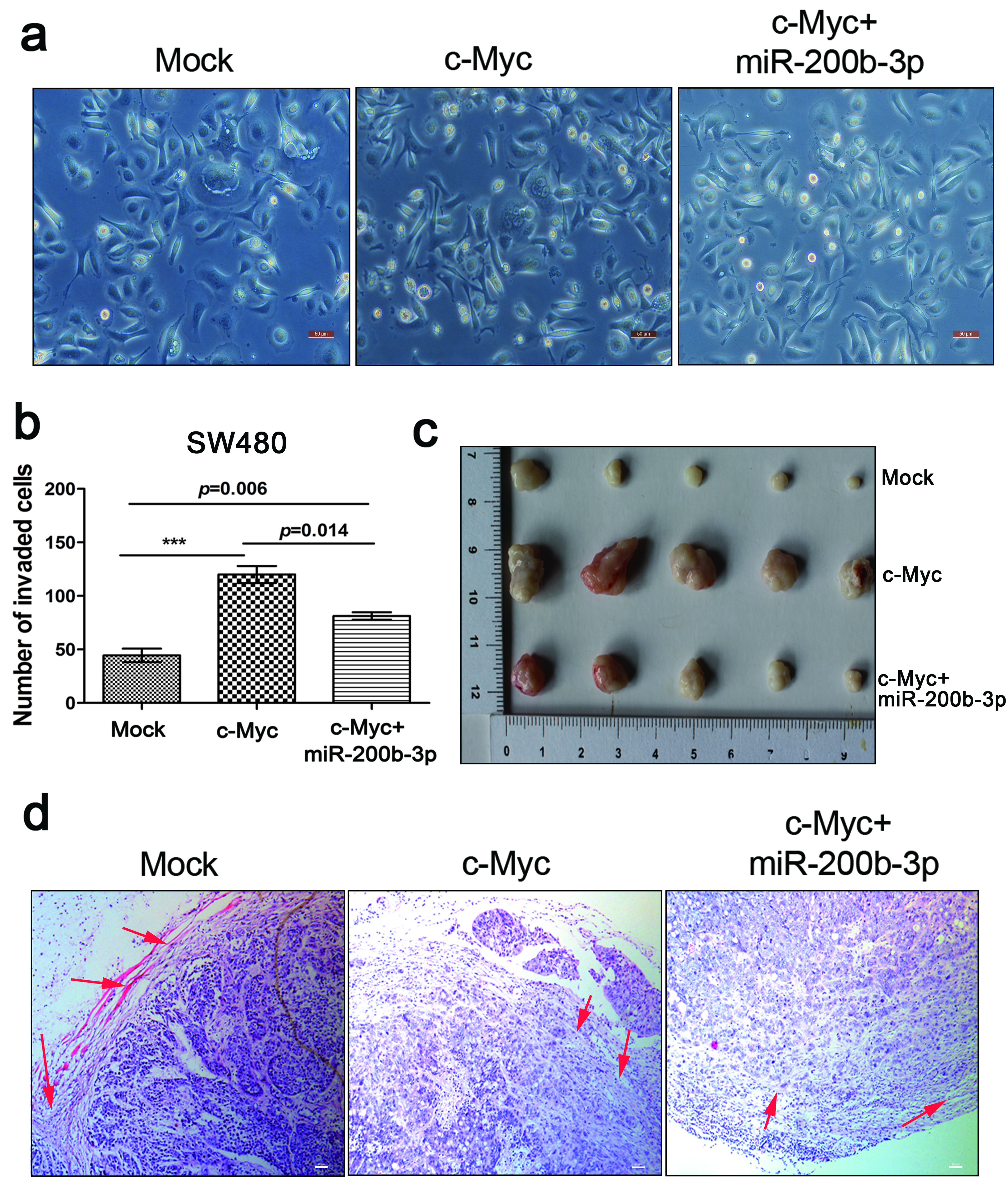

Supplement: Supplementary file 3 — Additional file 3: Figure S3. c-Myc represses growth, invasion and EMT of CRC cell by regulating miR-200b-3p. (a) Morphological changes of SW480 cells were observed under the microscope after overexpression of c-Myc and co-expression of c-Myc and miR-200b-3p. Scale bars represent 50 μm. (b) The number of invaded cells was counted under the microscope, with five HPF observation (*** p < 0.001). (c) SW480/Mock, SW480/c-Myc and sw480/c-Myc+miR cells (1 × 10^6^) were subcutaneously injected into the nude mice (n = 5) for four weeks and the isolated subcutaneous tumors was observed with naked eyes. (d) HE staining for local invasion of subcutaneous tumors derived from SW480/Mock, SW480/c-Myc and SW480/c-Myc+miR cells. Red arrows point at false fibrous membrane. Scale bars represent 50 μm. [file 12967_2017_1357_MOESM3_ESM.tif]

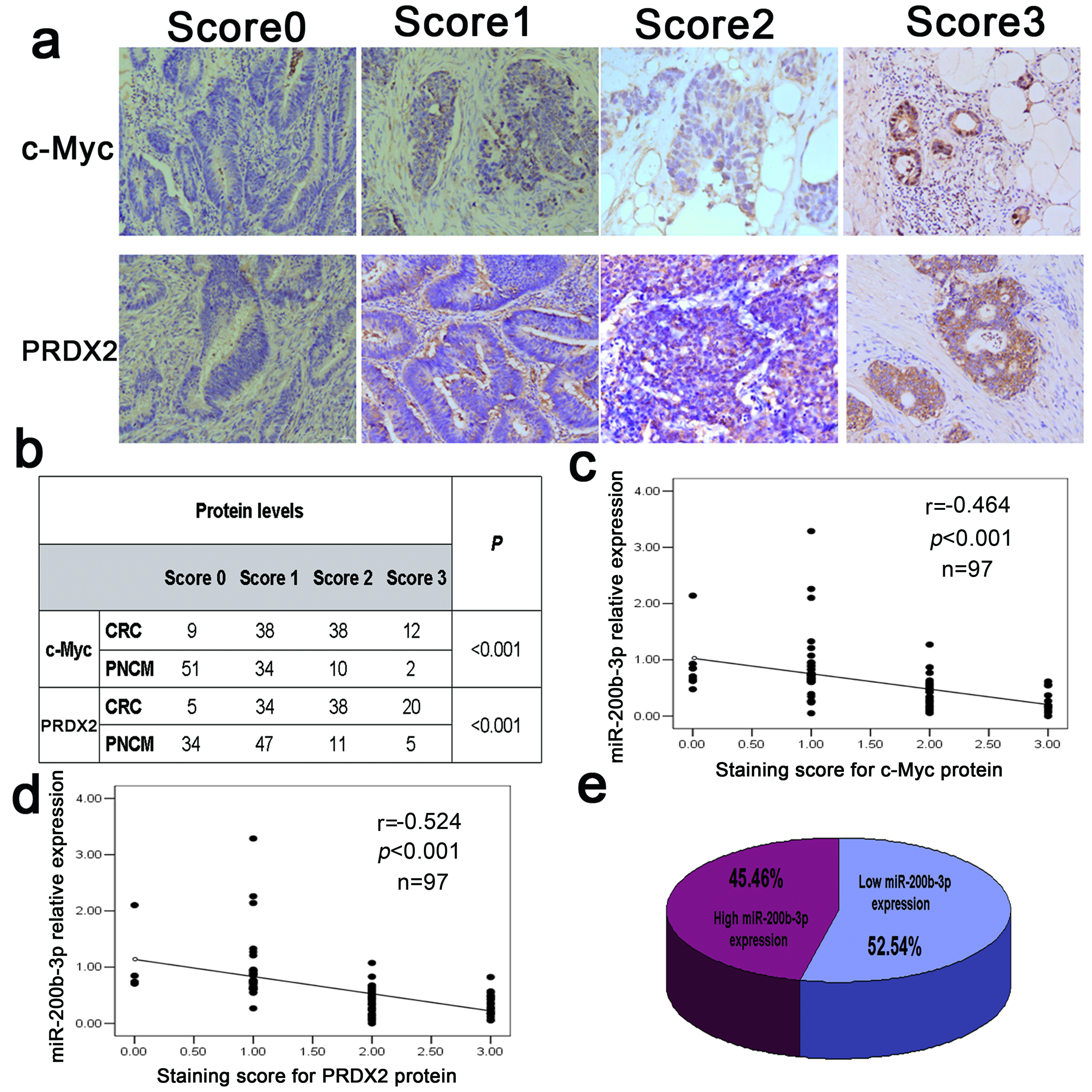

Supplement: Supplementary file 4 — Additional file 4: Figure S4. MiR-200b-3p is inversely correlated with c-Myc and PRDX2. (a) IHC staining for c-Myc and PRDX2 protein in CRC tissue samples. The c-Myc protein is mainly expressed in cell nucleus, whereas PRDX2 in cytoplasm. The scores (0, 1, 2 and 3) of the c-Myc and PRDX2 are based on their staining extents. (b) c-Myc and PRDX2 protein expression levels were frequently upregulated in CRC tissues compared to in PNCM tissues. (c, d) Inverse correlation of miR-200b-3p expression with c-Myc protein level (c), and with PRDX2 protein level (d) in CRC tissues. (e) The percentage of low and high miR-200b-3p expression in 97 cases of CRC tissues was presented in the pie chart. [file 12967_2017_1357_MOESM4_ESM.tif]
