# Supplementary material for: Disruption of the c-Myc/miR-200b-3p/PRDX2 regulatory loop enhances tumor metastasis and chemotherapeutic resistance in colorectal cancer
Source: J Transl Med. 2017 Dec 19;15:257. doi: 10.1186/s12967-017-1357-7 (PMC5735915; doi:10.1186/s12967-017-1357-7)
Supplement: Supplementary file 5 — Additional file 5: Table S1. The expression levels of c-Myc, PRDX2 and miR-200b-3p correlate with disease phenotypes of CRC patients. Table S2. Primer sequences for PCR amplification. [file 12967_2017_1357_MOESM5_ESM.doc]

Table S1: The expression levels of c-Myc, PRDX2 and miR-200b-3p correlate with disease phenotypes of CRC patients.

| **Variable** | **Cases** | **c-Myc protein expression** | | **miR-200b-3p expression** | | **PRDX2 protein expression** | |
| --- | --- | --- | --- | --- | --- | --- | --- |
|  |  | **staining extend scores（mean±SD）** | ***p*-value** | **Relative expression**  **(normalized to PNCM*)**  **（mean±SD）** | ***p*-value** | **staining extend scores**  **（mean±SD）** | ***p*-value** |
|  | 97 |  |  |  |  |  |  |
| **Age**  <60  ≥60 | 56  41 | 1.571±0.850  1.512±0.810 | 0.730 | 0.587±0.517  0.624±0.457 | 0.717 | 1.839±0.491  1.634±0.306 | 0.238 |
| **Gender**  Male  Female | 64  33 | 1.531±0.816  1.576±0.867 | 0.804 | 0.646±0.535  0.512±0.383 | 0.229 | 1.672±0.856  1.909±0.805 | 0.190 |
| **Differentiation**  Well  Moderate  Poor | 20  53  24 | 1.300±0.865  1.528±0.890  1.792±0.588 | 0.143 | 0.930±0.762  0.537±0.366  0.473±0.315 | 0.002 | 1.150±0.587  1.774±0.776  2.208±0.884 | 0.000 |
| **Tumor Size**  <5cm  ≥5cm | 57  40 | 1.316±0.827  1.875±0.723 | 0.001 | 0.731±0.563  0.420±0.279 | 0.002 | 1.386±0.750  2.275±0.679 | 0.000 |
| **pT-stage^+^**  T0-2  T3-4 | 26  71 | 1.269±0.962  1.648±0.758 | 0.046 | 0.833±0.702  0.518±0.355 | 0.037 | 1.423±0.945  1.873±0.773 | 0.019 |
| **pN-stage^++^**  N0  N1-2 | 52  45 | 1.212±0.848  1.933±0.618 | 0.000 | 0.777±0.562  0.401±0.285 | 0.000 | 1.327±0.760  2.244±0.645 | 0.000 |
| **pM-stage^+++^**  M0  M1 | 82  17 | 1.425±0.823  2.118±0.600 | 0.000 | 0.687±0.497  0.204±0.136 | 0.000 | 1.586±0.807  2.529±0.514 | 0.000 |

^*^ Paired normal colon mucosa. ^+^ Pathological Tumor stage. ^++^Pathological Node stage. ^+++^Pathological Metastasis stage

Table S2: Primer sequences for PCR amplification

| Gene primers sequences (5’-3’) |
| --- |
| miR-200b-3p promoter Forward AGCTGCAAGGGACGAGTG  (R1) Reverse GCCACCAATGCCATCAAA  miR-200b-3p promoter Forward GGTGCCAGAAAACTTGAAGAG  (R2) Reverse CACTCAGGACCCGAGGGA  miR-200b-3p promoter Forward AGCTGCAAGGGACGAGTG  (R3) Reverse GCCACCAATGCCATCAAA |
